# Unveiling the Anti-Aging Potential of 3HB: Lifespan Extension and Cellular Senescence Delay

**DOI:** 10.3390/nu17101647

**Published:** 2025-05-12

**Authors:** Yongpan An, Qian Wang, Panshuang Qiao, Jihan Liu, Ang Ma, Yutong Chen, Daqian Yang, Yi Ying, Nannan Li, Feng Lu, Hang Zhang, Guoqiang Chen, Yinhua Zhu, Baoxue Yang, Zhengwei Xie

**Affiliations:** 1 Peking University International Cancer Institute, Health Science Center, Peking University, 38 Xueyuan Lu, Haidian District, Beijing 100191, China; anyongpan123@163.com (Y.A.); 2211110101@stu.pku.edu.cn (Q.W.); cyt18304335821@163.com (Y.C.); yangdaqian9206@163.com (D.Y.); 2 Department of Pharmacology, School of Basic Medical Sciences, Peking University, 38 Xueyuan Lu, Haidian District, Beijing 100191, China; panshuangqiao@bjmu.edu.cn (P.Q.); liujihan@stu.pku.edu.cn (J.L.); yingyi1125@163.com (Y.Y.); 1610305107@pku.edu.cn (N.L.); fenglu202110@163.com (F.L.); hangzhang@bjmu.edu.cn (H.Z.); 3 State Key Laboratory for Quality Ensurance and Sustainable Use of Dao-di Herbs, Artemisinin Research Center, Institute of Chinese Materia Medica, China Academy of Chinese Medical Sciences, Beijing 100700, China; ama@icmm.ac.cn (A.M.); zhuyinhua@cau.edu.cn (Y.Z.); 4 School of Life Sciences, Tsinghua University, Beijing 100084, China; chengq@tsinghua.edu.cn; 5 State Key Laboratory of Natural and Biomimetic Drugs, Department of Molecular and Cellular Pharmacology, School of Pharmaceutical Sciences, Peking University Health Science Center, 38 Xueyuan Lu, Haidian District, Beijing 100191, China; 6 Peking University—Yunnan Baiyao International Medical Research Center, Peking University Health Science Center, Peking University, 38 Xueyuan Lu, Haidian District, Beijing 100191, China

**Keywords:** 3HB, aging, cellular senescence, metabolism

## Abstract

Background/Objective: Aging is a significant risk factor for chronic diseases and disability, yet effective anti-aging interventions remain elusive. We explored the potential of 3-hydroxybutyrate (3HB), an endogenous metabolite with established safety, to modulate longevity in mice. Methods: In this study, we employed 2BS and WI-38 cell models, a yeast model, and naturally aging mouse models to investigate the effects of 3HB on aging in various systems. Additionally, we utilized RNA sequencing and metabolomics technologies to explore the potential mechanisms underlying the action of 3HB. Results: Our findings demonstrate that 3HB supplementation effectively delays cellular senescence, extending yeast lifespan by 51.3% and the median lifespan of naturally senescent mice by 21.0%. Notably, 3HB prolonged healthy lifespan in mice while mitigating age-related tissue morphology changes and organ senescence. Mechanistically, we identified that 3HB’s anti-aging properties are mediated through its ability to delay cellular senescence and metabolic reprogramming, while promoting the production of beneficial metabolites like trigoneline and isoguvacine. Conclusions: These findings highlight the promising therapeutic potential of 3HB as an anti-aging intervention and provide novel insights into its underlying mechanisms.

## 1. Introduction

Human aging, a complex biological tapestry woven from declining physiological function, diminished resilience, and heightened susceptibility to disease, poses a growing challenge as our population ages rapidly. Globally, the over 65 demographic outpaces all others, with the United Nations predicting one in six people will exceed 65 by 2050, while the over 80 population triples. This demographic shift intensifies the urgency of unraveling aging’s intricate mechanisms, as its shadow looms large over chronic diseases like cardiovascular pathologies, cancer, and neurodegenerative disorders [1,2]. Understanding how to decelerate aging and compress its associated maladies thus emerges as a pivotal quest for our society.

Driven by the desire to extend lifespans and curb the tide of age-related diseases, the past decade has witnessed a surge in anti-aging strategies. From regenerative medicine and gene therapy to dietary tweaks, like ketogenic diets and calorie restriction, the scientific community has explored diverse avenues. However, while dietary and lifestyle interventions face challenges in adherence and long-term sustainability, more invasive approaches, like regenerative medicine and gene therapy, remain largely experimental and ethically complex. This has thrust the development of aging intervention drugs to the forefront, offering a potentially less intrusive and scalable solution. Anti-aging drug candidates, like dasatinib + quercetin [3], β-nicotinamide mononucleotide (NMN) [4], metformin [5,6], oridonin [7], Alpha-Ketoglutarate [8], berberine [9], and rapamycin [10,11], have shown promise, yet their clinical translation requires extensive research and rigorous safety assessments. The need for safer, easier-to-apply compounds with comparable efficacy remains, calling for continued innovation in this burgeoning field.

Emerging as a promising candidate in the anti-aging arsenal is 3-hydroxybutyrate (3HB), a naturally occurring metabolite with a favorable safety profile. Derived from human ketone bodies, 3HB boasts a wide range of safe doses. Its precursor, 1,3-butanediol (1,3-BDO), readily converts to 3HB in the liver, fueling bodily functions and holding a recognized status as a safe food additive [12]. Beyond its established uses, compelling evidence points towards 3HB’s therapeutic potential in diverse age-related ailments, like atherosclerosis [13], diabetes [12], muscular dystrophy [14], colitis [15], and kidney-related diseases [16]. 3HB’s influence extends beyond specific diseases, impacting vascular aging and even demonstrating lifespan extension in fruit flies and nematodes [17,18,19]. Intriguingly, the ketogenic diet, known to elevate 3HB levels, also exhibits lifespan-extending effects in mice, suggesting a potential link between 3HB and the diet’s longevity benefits [20,21]. However, a crucial question remains: can direct 3HB supplementation replicate these lifespan-extending effects in mammals and enhance their healthy lifespan?

Cellular senescence and metabolic dysregulation are hallmarks of aging, making their modulation key targets for anti-aging interventions. As an endogenous metabolite with established safety due to its precursor’s use as a food additive, 3HB holds promise. Studies have shown its ability to improve metabolic health in mice alongside a ketogenic diet. However, its impact on cellular senescence and specific lifespan-extending metabolite changes remained unclear.

We addressed these crucial gaps by demonstrating that 3HB directly delays cellular senescence, extends lifespans in both yeast and mice (including healthy lifespan), and mitigates age-related tissue and organ decline. Mechanistically, this anti-aging effect appears to involve both senescence delay and the promotion of beneficial metabolites like trigoneline and isoguvacine. Notably, 3HB also presents cost advantages due to its availability from microbial poly-(3-hydroxybutyrate) [22]. These findings establish 3HB as a promising anti-aging candidate with a well-tolerated precursor, providing both practical and theoretical value for future research and potential applications.

## 2. Materials and Methods

### 2.1. Drug Source

Since 3HB is stably preserved as 3HB-Na, in this experiment, we all utilized 3HB-Na for the experiments, which was obtained from Chen lab Tsinghua University, Beijing, China. Trigonelline and isoguvacine were all purchased from TargetMol, Boston, MA, USA.

### 2.2. Cell Lines and Cell Culture

WI-38 and 2BS cells were provided by the National Institutes for Food and Drug Control. We counted the cells and inoculated each passage at the same density, supplemented with 10% FBS in an incubator at 37 °C and 5% CO_2_ (Gibco, London, UK) in MEM (Gibco).

### 2.3. Cell Viability Test and Growth Analysis

The cell viability was evaluated by the CCK-8 detection kit (Dojindo, Kumamoto, Japan). The 2BS or WI-38 cells were seeded into 96-well plates (5000/well) and then treated with drugs for 24 h. A 1:10 diluted CCK-8 solution was added to the culture medium and incubated for 1 h at 37 °C. Then a microplate reader (Biotek, Winooski, VT, USA, MQX200) was used to measure the absorbance at 450 nm.

Cell survival rate (%) = (OD treatment − OD_blank_)/(OD_control_ − OD_blank_) × 100%

The CCK-8 kit is also used as a method for assessing cell proliferation by plating cells (2000/well) in 96-well plates. Then the cells were treated with drugs for 1 week. We refreshed the medium every day with fresh drugs or DMSO. At an appropriate time point, we treated them with CCK-8 solution for 1 h as described above, and measured the absorbance at 450 nm.

### 2.4. Senescence-Associated β-Galactosidase (SA-β-Gal) Staining

We washed the cells in PBS, then fixed them with 4% formaldehyde at room temperature for 5 min, and then washed them again. Then cells were stained with a buffer (1 mg/mL 5-bromo-4-chloro-3-indolyl-β-d-galactopyranoside (X-gal), 40 mM citric acid at 37 °C/Sodium phosphate, pH 6.0) Stain overnight, 5 mM potassium ferrocyanide, 5 mM potassium ferricyanide, 150 mM NaCl, 2 mM MgCl_2_). The cells were then analyzed according to the instructions provided (CST, Danvers, MA, USA, 9860S).

### 2.5. Yeast Mutant Generation

Wild-type yeast all came from the library from Krogen lab (UCSF), San Francisco, CA, USA. Standard SD medium (1% yeast extract, 1% bacterial protein, 2% glucose) was used for routine growth of all yeast strains on a rotary shaker at 250 rpm at 30 °C. The experiments designed to assess the lifespan of the yeast were carried out with the yeast in the logarithmic growth phase by culturing the yeast overnight and then diluting it 4 h before use.

### 2.6. Yeast Replication Lifespan Test

As mentioned in previous reports, we derived a modified U-shape. Due to our work on berberine [9], we used a high-pressure microfluidic chip, which resulted in a slightly shorter yeast lifespan, but the effects of lifespan extension in yeast, lifespan extension in human senescent cells, and lifespan extension in mice treated with various compounds were all consistent. We observed mother cells for two days using time-lapse microscopy. This method relies on U-shaped wells in a microfluidic system to keep the mother cells in place while allowing the flowing medium to carry away the daughter cells. Survival curves were plotted based on data collected from multiple experiments. We plotted survival curves and cell cycle curves using Matlab 2018a.

### 2.7. Animals and Survival Curve

All young and naturally aged mice were purchased from SPF Biotechnology Co., Ltd., Beijing, China. The mice were kept in an environment with minimum pressure and standard conditions (constant temperature and light/dark at 12:12 h). The cage size was 325 mm × 210 mm × 150 mm. The cages were made of plastic and were lined with corn cob bedding. Each cage housed four mice. Animals were randomly assigned to control and treatment groups. We purchased 8 ICR mice at the age of 2 months, with an average weight of 35 g. We purchased 69 ICR mice at the age of 11 months, with an average weight of 47 g.

Survival curves: Before grouping, the weight of each mouse was measured, and they were randomly divided into groups according to their weight. All mice were included in the experiment, with no mice excluded. We selected 11-month-old male ICR mice, dissolved 3HB-Na in drinking water (1 g/L), and administered 3HB-Na to 11-month-old mice in drinking water until the mice died naturally, and recorded the body weights and survival conditions.

### 2.8. Grip Test

The mice were placed on top of the grid strength meter, so they grabbed the grid with all four paws. The grip strength of 5 trials was recorded, and the final data were taken as the maximum grip strength (g).

### 2.9. Physical Appearance and Posture Score

Physical appearance and posture scores were performed on 2- and 20-month-old ICR mice [23]. Physical appearance: 0-normal, 1-lack of grooming, 2-rough hair coat, 3-very rough hair coat. Posture: 0-normal, 1-sitting in hunched position, 4-hunched posture, head resting on floor, 6-lying prone on cage floor/unable to maintain upright posture. The score is the sum of the two.

### 2.10. Tissue Morphology Staining and Statistics

Male ICR mice were studied in a young control group (2 months of age), an old control group, and an old treatment group (20 months of age), and 3HB (1 g/L) was administered in the form of drinking water from 11 months of age. The mice were euthanized by cervical dislocation performed by personnel who had undergone professional training. The hearts, livers, kidneys, and muscles of the mice were taken.

Masson’s trichrome stain: To test fibrosis of heart tissue, Masson’s trichrome stain kit (Solarbio, Beijing, China, G1340) was used. The tissue was fixed with 4% formaldehyde, followed by paraffin embedding and sectioning. The sections were then stained according to the manufacturer’s instructions. The slices were quickly dehydrated with 95% alcohol and 100% alcohol, cleaned with xylene, and then covered with a coverslip. Images were collected using a strong light microscope (20× magnification objective lens) (OLYMPUS, Tokyo, Japan, BX43) and analyzed by ImageJ 1.53a software (NIH, Bethesda, MD, USA) to measure the fibrosis area and the total area. Each organization used 5 different areas for measurement.

H & E staining: To test the morphological changes of tissues. The kidney, liver, brain, and muscle were fixed with 4% formaldehyde, followed by paraffin embedding and sectioning. The sections were stained with H & E for morphological analysis.

For renal tissue, when the tubular brush border was lost, the lumen was dilated, and the epithelial cells were flattened and atrophied, the damaged tubules were counted. The percentage of injured tubules to total tubules was calculated and scored according to the following criteria: 0 = normal: 1 ≤ 20%; 2 = 20% to 40%; 3 = 40% to 60%; 4 = 60% to 80%; and 5 ≥ 80%. For the liver, the Knodell Histological Activity Index (HAI) was utilized for scoring. The system uses four independent criteria for scoring: (1) peripheral fragmentation necrosis (PN) with or without bridging necrosis (BN) in the confluent area (0–10 points), (2) hepatocellular degeneration and focal necrosis in the lobules (0~4 points), (3) inflammation in the confluent area (0~4 points), and (4) fibrosis (0~4 points), and the sum of the scores of each of the four indexes is the total score. For muscle, 100 muscle fibers were randomly selected from each mouse and muscle fiber area and feret were detected using imageJ.

### 2.11. RNA Extraction and qPCR

TRIzol was used to lyse cells, and then isopropanol and chloroform were used to extract RNA. Using cDNA synthesis kit, a total of 1 μg RNA was used for cDNA reverse transcription (Abmgood, Richmond, BC, Canada). Real-time qPCR was used to evaluate the target gene’s expression, and the expression level was normalized to ACTIN. Supplementary Table S1 lists all primers.

### 2.12. RNA-Seq

For animal handling, see tissue morphology staining. We performed RNA extraction and RNA-seq detection with ICR mice muscle tissue. Total RNA was extracted from the tissue using TRIzol^®^ Reagent according to the manufacturer’s instructions (Invitrogen, Waltham, MA, USA) and genomic DNA was removed using DNase I (TaKara, San Jose, CA, USA). Then, RNA quality was determined with a 2100 Bioanalyser (Agilent, Santa Clara, CA, USA) and quantified using the ND-2000 (NanoDrop Technologies, Wilmington, DE, USA). Only the high-quality RNA sample (OD260/280 = 1.8~2.2, OD260/230 ≥ 2.0, RIN ≥ 6.5, 28S:18S ≥ 1.0, >2 μg) was used to construct the sequencing library. After quantification with TBS380, the paired-end RNA-seq sequencing library was sequenced with the Illumina HiSeq xten/NovaSeq 6000 sequencer (2 × 150 bp read length). RNA sequencing and raw data quality control were performed by Shanghai Majorbio Bio-pharm Technology Co., Ltd., Shanghai, China.

### 2.13. Echocardiographic Measurements

We performed cardiac ultrasound in 2-month-old, 20-month-old, and 3HB-intervention 20-month-old male ICR mice. To measure cardiac physiological functions, an ultrasound cardiotachograph (VINNO 6 VET, VINNO, Suzhou, China) was used for the detection and analysis of echocardiography. B-mode ultrasonography was applied in seeking the long axis of left ventricular, then 2-dimensional M-mode was transferred for data acquisition. Left ventricle internal dimension diastole (LVIDd), left ventricle internal dimension systole (LVIDs), and ejection fraction (EF), among others, were directly measured by using M-modeanalysis. Fractional shortening (FS) was calculated by LVIDd and LVIDs.

### 2.14. Tissue SA-β-Gal Staining

Male ICR mice were studied in an old control group and an old treatment group (20 months of age), and 3HB (1 g/L) was administered in the form of drinking water from 11 months of age. The mice were euthanized by cervical dislocation performed by personnel who had undergone professional training. The hearts, livers, kidneys, and colons of the mice were taken, and frozen sections were prepared by liquid nitrogen flash freezing. Frozen sections were first rewarmed, washed with PBS 3 times (for at least 5 min each time), and fixed with an appropriate volume of β-galactosidase staining fixative to cover the tissue adequately (fixed for at least 15 min at room temperature). Wash the tissues with PBS immersion 3 times for at least 5 min each time. Aspirate the PBS and add an appropriate volume of staining work. Incubate overnight at 37 °C. The tissues were washed three times with PBS, stained with nuclear solid red for 1 min, and washed in water → anhydrous ethanol → xylene. Three randomly selected fields of view were photographed for each mouse and the blue area was counted using ImageJ 1.53a.

### 2.15. Serological Testing

For animal handling, see tissue morphology staining and statistics. For serum biochemical analysis, blood samples were collected, coagulated at room temperature for 2 h, or overnight at 4 °C, and then centrifuged (1000× *g*, 10 min) to obtain serum. A 200 μL aliquot of serum was taken and analyzed with a chemical analyzer (Mindray, Mahwah, NJ, USA, BS-350E) for uric acid (UA), blood urea nitrogen (BUN), alanine aminotransferase (ALT), aspartate aminotransferase (AST), creatine kinase isoenzymes (CK-MB), triglyceride (TG), total cholesterol (TCHO), High-Density Lipoprotein (HDL), and Low-Density Lipoprotein (LDL).

### 2.16. Metabolomics Assay

Feces were collected from 2-month-old, 20-month-old, and 3HB-intervention 20-month-old male ICR mice. Around 8 p.m., we placed the mouse on a sterile aluminum foil and allowed it to defecate naturally. We transferred the fecal pellets to a labeled sterile centrifuge tube using sterile forceps and then froze them rapidly in liquid nitrogen for storage at −80 °C. Samples were weighed before the extraction of metabolites and dried lyophilized were ground in a 2 mL Eppendorf tube containing a 5 mm tungsten bead for 1 min at 65 Hz in a Grinding Mill. Metabolites were extracted using 1 mL precooled mixtures of methanol, acetonitrile, and water (*v*/*v*/*v*, 2:2:1) and then placed for 1 h ultrasonic shaking in ice baths. Subsequently, the mixture was placed at −20 °C for 1 h and centrifuged at 14,000× *g* for 20 min at 4 °C. The supernatants were recovered and concentrated to dryness in vacuum.

UHPLC-MS/MS analysis: Metabolomics profiling was analyzed using a UPLC-ESI-Q-Orbitrap-MS system (UHPLC, Shimadzu Nexera X2 LC-30AD: Shimadzu, Japan) coupled with Q-Exactive Plus (Thermo Scientific, San Jose, CA, USA). For liquid chromatography (LC) separation, samples were analyzed using a ACQUITY UPLC^®^ HSS T3 column (2.1 × 100 mm, 1.8 μm) (Waters, Milford, MA, USA). The flow rate was 0.3 mL/min and the mobile phase contained the following: A, 0.1% FA in water and B, 100% acetonitrile (ACN). The gradient was 0% buffer B for 2 min and was linearly increased to 48% in 4 min, and then up to 100% in 4 min and maintained for 2 min, and then decreased to 0% buffer B in 0.1 min, with a 3 min re-equilibration period employed.

Data preprocessing and filtering: The raw MS data were processed using MS-DIAL for peak alignment, retention time correction, and peak area extraction. The metabolites were identified by accuracy mass (mass tolerance < 10 ppm) and MS/MS data (mass tolerance < 0.02 Da), which were matched with HMDB, massbank and other public databases and our self-built metabolite standard library. In the extracted-ion features, only the variables having more than 50% of the nonzero measurement values in at least one group were kept. Multivariate statistical analysis: R (version:4.0.3) and R packages were used for all multivariate data analyses and modeling. Data were mean-centered using Pareto scaling. Models were built on principal component analysis (PCA), orthogonal partial least-square discriminant analysis (PLS-DA), and partial least-square discriminant analysis (OPLS-DA). All the models evaluated were tested for over fitting with methods of permutation tests.

KEGG Enrichment analysis: To identify the perturbed biological pathways, the differential metabolite data performed the KEGG pathway analysis using the KEGG database (http://www.kegg.jp, accessed on 20 June 2022). KEGG enrichment analyses were carried out with the Fisher’s exact test, and FDR correction for multiple testing was performed. Enriched KEGG pathways were nominally statistically significant at the *p* < 0.05 level.

### 2.17. Statistics

Results were assessed by two-tailed Student’s t tests or ANOVAs to compare two and more than two samples, respectively. Data with means ± sem *p* < 0.05 was the significance threshold. * *p* < 0.05, ** *p* < 0.01, *** *p* < 0.001. Ns, not significant. Unless otherwise stated, the results are based on the results of at least three independent experiments. Unless otherwise stated, GraphPad Prism (Version 8.0.2) was used for statistics.

## 3. Results

### 3.1. 3HB Delays Cellular Senescence and Extends the Lifespan of Yeast

**Figure 1:**
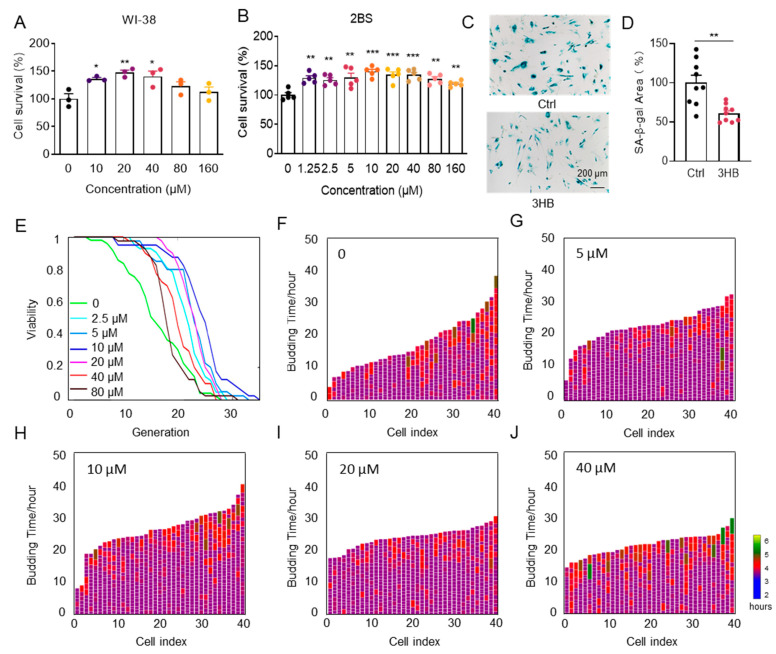
3HB delays cellular senescence and extends the lifespan of yeast. (**A**) The effect of different concentrations of 3HB on the proliferation of replicatively senescent WI-38 cells was measured by the CCK-8 assay. (**B**) The effect of different concentrations of 3HB on the proliferation of replicatively senescent 2BS cells was measured by the CCK-8 assay. (**C**) SA-β-gal staining of replicatively senescent 2BS cells treated with 10 µM 3HB or DMSO for 48 h. (**D**) Quantification of the rate of SA-β-gal positive cells in replicatively senescent 2BS cells. (**E**) 3HB extends the replication lifespan of yeast. (**F**–**J**) Germination diagrams of wild-type and 3HB-treated mother cells show cell cycle duration and heterogeneity (*n* = 40 for each group). (See index color scale. Duration is 1.4 h or less. Cell cycle is colored in the color purple). The *x*-axis shows a single parent cell as a vertical bar, while budding events are shown as white horizontal partitions. Data represent the mean ± SEM. *p* values were determined by one-way ANOVA, two-way ANOVA, or Student’s *t*-test. (* *p* < 0.05, ** *p* < 0.01, *** *p* < 0.001).

To investigate if 3HB can delay cellular senescence in human cell lines, we treated both senescent human embryonic lung fibroblasts WI-38 and 2BS (PD45) with different concentrations of 3HB. 3HB significantly increased the viability of senescent cells in both cell lines in a dose-dependent manner (Figure 1A,B). Importantly, 3HB treatment not only enhanced cell viability but also alleviated morphological hallmarks of senescence, such as granular cytoplasm and inclusion body accumulation. Consistent with these observations, SA-β-gal staining, a marker of senescence, was significantly reduced in 3HB-treated 2BS cells compared to aged controls (38.8% reduction, Figure 1C,D). Simultaneously, the intervention of 3HB reduced the expression levels of p53, p21, IL-6, IL-1β, and CXCL2 in senescent 2BS cells (Figure S1A). Altogether, these findings suggest that 3HB effectively maintains the viability of senescent cells, slows down the replicative senescence process, and holds promise as a potential anti-aging intervention.

While developing anti-aging drugs is notoriously difficult, long-term lifespan testing in model organisms remains crucial for identifying promising candidates. Saccharomyces cerevisiae, a single-celled fungus commonly used in aging research, provides a valuable window into human aging due to its highly conserved aging mechanisms with mammals [24]. To accurately assess the replicative lifespan of yeast in this study, we utilized an automated device based on a microfluidic chip [25], capable of measuring lifespan within 3 days. Yeast incubated in SD medium for 20 h were then collected and loaded onto the chip. Lifespan was monitored at 3HB concentrations ranging from 0 to 80 μM. Our results demonstrated a dose-dependent prolongation of the replicative lifespan of the BY4741 strain compared to the control, with 10 µM of 3HB leading to a remarkable 51.3% extension (Figure 1E). Notably, 3HB treatment also significantly reduced cell cycle duration (Figure 1F–J and Figure S1B,C), reducing its heterogeneity and further contributing to chronological lifespan extension. These findings strongly suggest 3HB’s potential as a promising anti-aging intervention.

### 3.2. Treatment with 3HB Significantly Extended Lifespan by 123 Days and Improved Healthspan as Measured by Increased Physical Activity

**Figure 2:**
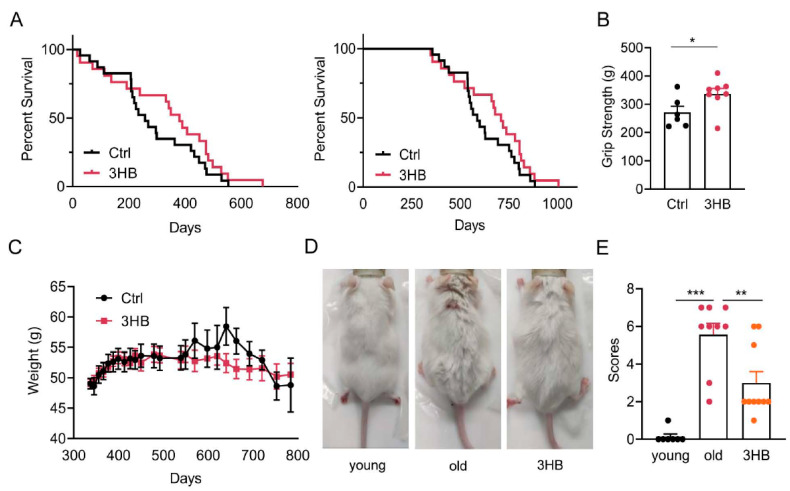
3HB extends the lifespan of mice. (**A**) Lifespan curves of 11-month-old ICR mice under 3HB intervention from the beginning of treatment (left) and from birth (right) (Ctrl *n* = 23, 3HB *n* = 21). (**B**) Effect of 3HB on grip strength of 20-month-old ICR male mice (*n* ≥ 6). (**C**) Changes in body weight of ICR mice under 3HB intervention (*n* ≥ 6). (**D**,**E**) Effects of 3HB on hair and body size of 20-month-old male ICR mice and statistics (*n* ≥ 6). Data represent the mean ± SEM. *p* values were determined by one-way ANOVA, two-way ANOVA, or Student’s *t*-test. (* *p* < 0.05, ** *p* < 0.01, *** *p* < 0.001).

Based on the aforementioned observations, we posit that 3HB has the potential to extend the lifespan of mice. To verify this hypothesis, we designed an in vivo experiment where 11-month-old ICR male mice were divided equally into two groups: a control group and a 3HB-treated group. We ensured that there was no significant difference in initial body weight and grip strength between the two groups (Figure S2A,B). Over the course of the experiment, we observed a remarkable extension in the median lifespan of the 3HB-treated group compared to the control group. The median (mean) lifespan of the 3HB-treated mice was extended by 123 (52) days (approximately 21.0% (8.4%)), increasing from 587 (618) days to 710 (670) days (Figure 2A).

Functional tests revealed that 3HB treatment significantly improved the grip strength (Figure 2B) and clinical scores (Figure 2D,E) of the aged mice compared to the control group. Notably, 3HB intervention also influenced the age-induced body weight changes. While the control group mice experienced an increase followed by a decrease in body weight as they aged, the 3HB-treated group displayed a more stable body weight pattern with a reduced amplitude of change (Figure 2C). Interestingly, there was no significant difference in dietary intake between the two groups (Figure S2C), suggesting that the observed effects were not due to differences in food consumption. The results of the cardiac ultrasound showed that 3HB had a tendency to improve all cardiac indices in aging mice, but there was no statistical difference (Figure S2D–L). Collectively, these findings strongly suggest that 3HB can extend the lifespan and improve the healthy lifespan of naturally aging mice.

### 3.3. 3HB Has the Potential to Improve Age-Related Histomorphometric Changes in Kidney and Muscle

**Figure 3:**
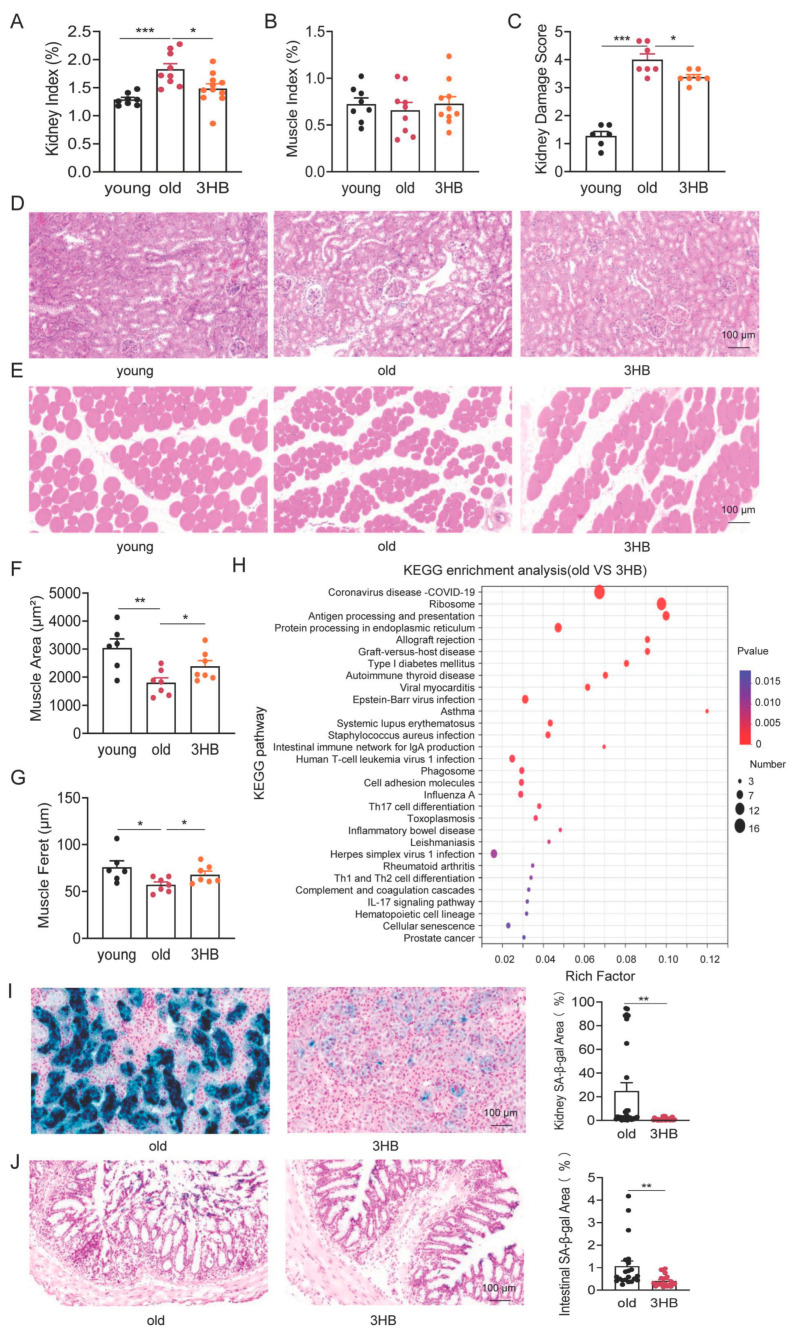
Effects of 3HB intervention on morphological changes and cellular senescence in mouse organ tissues. (**A**,**B**) Kidney and muscle organ index of male ICR mice at 2 months (young), 20 months (old), and 20 months treated with 3HB (3HB) (Organ weight/body weight * 100%). (**C**,**D**) HE staining to detect the histomorphology of kidney of male ICR mice at 2 months (young), 20 months (old), and 20 months treated with 3HB (3HB), and their quantitative statistics. (**E**–**G**) HE staining to detect the histomorphology of muscle of male ICR mice at 2 months (young), 20 months (old), and 20 months treated with 3HB (3HB), and muscle fiber area and feret statistics. (**H**) KEGG enrichment analysis (kidney tissue) of signaling pathway changes due to 3HB intervention. (**I**) SA-β-Gal staining to detect the effect of 3HB intervention on the cellular senescence of kidney tissues in 20-month-old male ICR mice and statistics. (**J**) SA-β-Gal staining to detect the effect of 3HB intervention on the cellular senescence of colon tissue in 20-month-old male ICR mice and statistics. Data represent the mean ± SEM (*n* ≥ 6). *p* values were determined by one-way ANOVA, two-way ANOVA, or Student’s *t*-test. (* *p* < 0.05, ** *p* < 0.01, *** *p* < 0.001).

During aging, the senescence of individual cells leads to reduced tissue and organ function as well as morphological changes [26]. To investigate how 3HB might counteract these declines, we analyzed major organs in young (2-month-old), aged (20-month-old), and aged mice treated with 3HB (20-month-old with 3HB intervention). We found that 3HB treatment notably ameliorated age-related changes in kidney function, including improved renal indices (Figure 3A) and trends towards better kidney function parameters like uric acid (UA), urea nitrogen (BUN), and increased muscle weight (Figure 3B and Figure S3A,B). We performed HE staining analysis on the kidney tissues of aged mice and 3HB-treated aged mice. The results showed that the glomerular structure was restored and glomerular atrophy was reduced in the mice treated with 3HB (Figure 3C,D). This suggests 3HB protects against aging-related kidney damage.

3HB also counteracted age-induced muscle changes, characterized by increased muscle fiber area and feret diameter (Figure 3E–G), indicating restored muscle health. While 3HB showed positive effects on kidney and muscle, it did not significantly impact liver, brain, heart, spleen, or thymus function or morphology (Figure S3C–N).

These findings suggest that 3HB has the potential to improve age-related histomorphometric changes in specific tissues, like kidney and muscle, warranting further research into its potential as an anti-aging intervention.

### 3.4. 3HB Combats Aging by Targeting Cellular Senescence in Kidney and Colon

To understand how 3HB exerts its lifespan and healthspan-promoting effects, we delved into gene expression changes in mouse kidneys. We performed RNA sequencing on kidney tissues from young (2-month-old), aged (20-month-old), and aged mice treated with 3HB (20-month-old with 3HB intervention). Analyzing differentially expressed genes between aged and 3HB-treated aged mice, as well as those intersecting with differentially expressed genes from young mice, revealed an enrichment of cellular senescence signaling pathways in both groups (Figure 3H and Figure S4A). This suggests that 3HB might act by influencing senescence processes. Focusing on senescence markers in various organs, we found that 3HB delayed cellular senescence in kidney and colon tissues (Figure 3I,J). However, 3HB did not show significant effects on cellular senescence in liver and heart tissues (Figure S4B–E).

### 3.5. 3HB Modulated Lipid Metabolism and Reversed Some Age-Related Metabolic Changes

**Figure 4:**
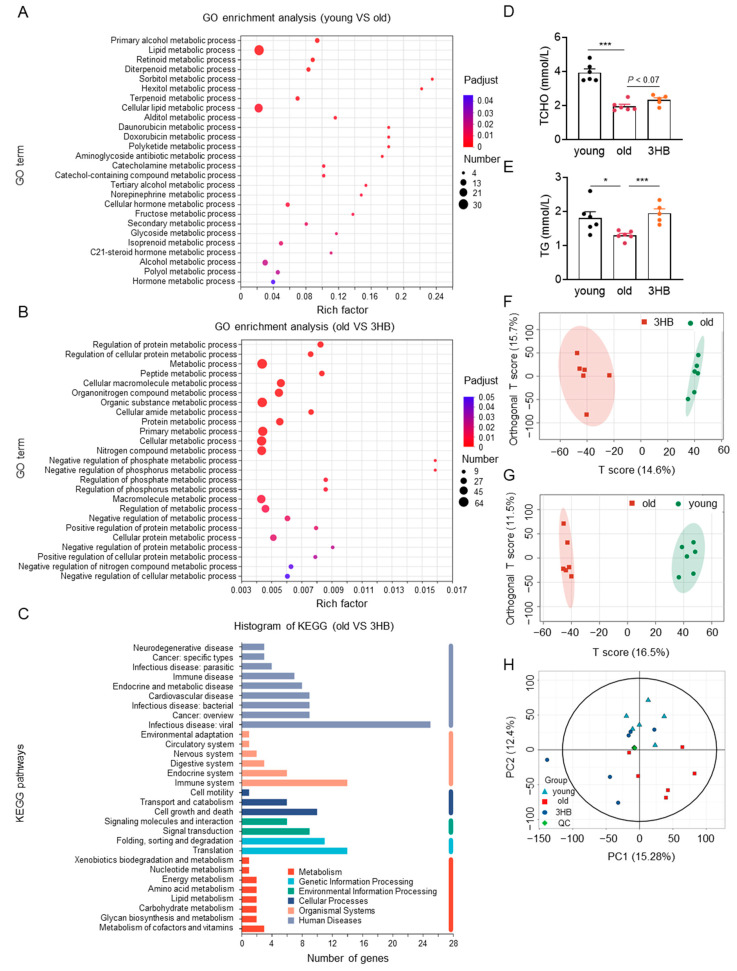
The influence of 3HB on metabolic changes of aged mice. (**A**) GO enrichment analysis (kidney tissue) of changes in metabolic signaling pathways due to 3HB intervention (*n* = 3). (**B**) GO enrichment analysis of genes for differences between the 3 groups (2-month-old, 20-month-old, and 20-month-old mice with 3HB intervention) demonstrated changes in metabolism-related signaling pathways (*n* = 3). (**C**) KEGG annotation analysis (kidney tissue) of changes in signaling pathways due to 3HB intervention (*n* = 3). (**D**,**E**) Blood biochemistry for total cholesterol (TCHO) and triglycerides (TG) in male ICR mice at 2 months (young), 20 months (old), and 20 months treated with 3HB (3HB). Metabolomics of feces from 20-month-old ICR mice at 2 months, 20 months (old), and 20 months treated with 3HB (3HB) (*n* ≥ 6). (**F**) PCA analysis (NEG) of differential metabolites in the aged and 3HB-intervened aged groups. (**G**) PCA analysis (NEG) of differential metabolites between the younger and older groups (*n* ≥ 6). (**H**) PCA analysis of differential metabolites between the three groups (NEG) (*n* ≥ 6). Data represent the mean ± SEM. *p* values were determined by one-way ANOVA, two-way ANOVA, or Student’s *t*-test. (* *p* < 0.05, *** *p* < 0.001).

Delving deeper into mechanisms, we performed GO and KEGG analyses on gene expression data, revealing a significant enrichment of metabolism-related signaling pathways across different groups (Figure 4A–C). This suggests 3HB might influence metabolic processes during its lifespan-extending effects. Focusing on lipid metabolism, we observed that 3HB reversed age-related increases in triglycerides and total cholesterol in mice (Figure 4D,E), potentially contributing to its health benefits. Notably, low-density lipoprotein (LDL) and high-density lipoprotein (HDL) levels remained unaffected (Figure S5A,B).

To gain further insights into metabolic changes, we performed metabolomic sequencing on fecal samples from young, aged, and 3HB-treated aged mice. Principal component analysis (PCA) revealed distinct metabolic profiles for each group and highlighted the impact of both aging and 3HB intervention (Figure 4F,G and Figure S5C,D). Additionally, PCA within the 3 groups showed that 3HB treatment shifted some aged mice closer to the young mouse metabolic profile (Figure 4H and Figure S5E–G).

These combined findings suggest that 3HB exerts its lifespan-extending effects through diverse metabolic pathways, with a notable modulation of lipid metabolism and a potential reversal of some age-related metabolic changes.

### 3.6. Trigonelline and Isoguvacine Might Contribute to 3HB’s Anti-Aging Effects by Reducing Cellular Senescence

**Figure 5:**
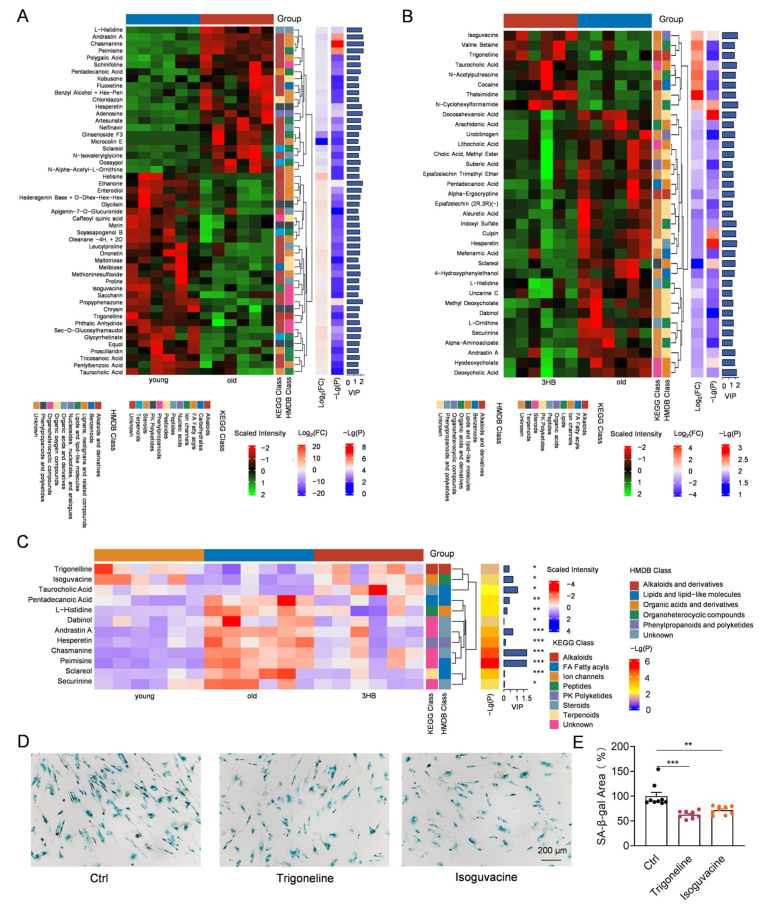
3HB affects cellular senescence by altering metabolites in mice. (**A**) Heatmap analysis of metabolite changes in young and senescent mice (*n* ≥ 6). (**B**) Heatmap analysis of changes in metabolites due to 3HB intervention (*n* ≥ 6). (**C**) Heatmap analysis of differential metabolites between 3 groups (2-month-old, 20-month-old, and 20-month-old male ICR mice with 3HB intervention) (*n* ≥ 6). (**D**) SA-β-gal staining of replicatively senescent 2BS cells treated with trigoneline (10 µM), Isoguvacine (10 µM) or DMSO for 48 h. (**E**) Quantification of the rate of SA-β-gal positive cells in replicatively senescent 2BS cells. Data represent the mean ± SEM. *p* values were determined by one-way ANOVA, two-way ANOVA, or Student’s *t*-test. (* *p* < 0.05, ** *p* < 0.01, *** *p* < 0.001).

To delve deeper into 3HB’s metabolic influence, we analyzed differential metabolites between young and aged mice, revealing over 50 significantly altered compounds, including histidine, trigonelline, and taurocholic acid (Figure 5A). KEGG enrichment analysis linked these changes to pathways, like taurine and hypotaurine metabolism, carbohydrate digestion, and lipolysis regulation, suggesting broad metabolic reprogramming during aging (Figure S6A).

Similarly, comparing aged and 3HB-treated mice identified over 30 differentially expressed metabolites, including valine betaine and cocaine (Figure 5B). KEGG analysis linked these changes to similar pathways, like taurine metabolism and lipolysis regulation, but also highlighted cholesterol metabolism and central carbon metabolism (Figure S6B). This suggests 3HB might specifically target aspects of lipid metabolism alongside broader metabolic modulation.

Zooming in further, we focused on metabolites reversed by 3HB treatment in aged mice. Twelve compounds showed significant changes, including trigonelline and isoguvacine (Figure 5C). Notably, both trigonelline and isoguvacine are down-regulated in aged mice and up-regulated in young and 3HB-treated mice. Investigating their impact on cellular senescence, we found that both compounds reduced SA-β-gal positive cells, suggesting anti-senescence potential (Figure 5D,E).

Overall, this deep dive into differential metabolites reveals that 3HB induces broad metabolic changes in aged mice, potentially targeting lipolysis and cholesterol metabolism. Furthermore, specific metabolites, like trigonelline and isoguvacine, might contribute to 3HB’s anti-aging effects by reducing cellular senescence.

## 4. Discussion

This study unveils the promising anti-aging potential of 3HB, an endogenous metabolite. We observed remarkable benefits in aged mice: improved senescent cell viability, delayed cellular senescence, extended lifespan and healthspan, and restored health markers in kidney and muscle tissues. Notably, 3HB specifically countered senescence in the kidney and colon, further supporting its rejuvenating effects.

Delving deeper, our analyses suggest two key mechanisms: combating cellular senescence and modulating metabolic pathways. Sequencing data revealed that 3HB upregulated metabolites, like trigonelline and isoguvacine, potentially contributing to its lifespan-extending effects. These intriguing findings provide novel evidence for 3HB’s application in promoting healthy aging and warrant further investigation into its precise mechanisms of action.

While promising anti-aging candidates, like senolytics and mTOR inhibitors, exist, concerns regarding safety and clinical translation due to side effects remain [27,28,29]. Endogenous metabolites, like 3HB, offer a safer alternative due to their natural presence in the body. In this study, we found that 3HB intervention extended the median lifespan of mice by 21% (123 days) and the mean lifespan by 8.4% (52 days) throughout their entire life cycle (as shown in Figure 2A). We then compared 3HB with other known compounds that delay aging. Our findings revealed that metformin can extend the mean lifespan of mice by 4.15% from the start of the intervention [6]; NMN/NR can extend the mean lifespan of mice by 30.2% from the start of the intervention, and the mean lifespan throughout the entire life cycle can be extended by 4.7% [4]; rapamycin can extend the mean lifespan throughout the entire life cycle by 7% to 16% [10,11]; dasatinib + quercetin can extend the mean lifespan of mice by 36% from the start of the intervention [3]. From these studies, we can see that the anti-aging effects of 3HB are comparable to or even more outstanding than those of these well-known anti-aging compounds. Its established production process and the safe use history as a food additive (through its precursor) further solidify its potential. 3HB stands out with its unique combination of safety, efficacy, and applicability.

Beyond lifespan extension, 3HB ameliorated aging-related damage in the kidney and muscle, suggesting it improves healthy lifespan alongside longevity. This raises the possibility of using 3HB to treat age-related diseases like atherosclerosis [13,30], diabetes [31], and muscle loss [32]. While the ketogenic diet offers similar benefits, its dietary challenges limit its clinical practicality [33]. Direct 3HB supplementation may be a more viable option. This study adds to the growing evidence for 3HB’s role in treating age-related diseases.

In addition, we observed that 3HB improved cellular senescence in kidney and intestinal tissues but had no effect on cellular senescence in liver and heart tissues. Previous studies have indicated that the liver is the primary site for the synthesis of 3HB, but the liver itself utilizes relatively little 3HB. 3HB can cross the blood–brain barrier and be used by the brain as an energy source, especially during fasting or low-carbohydrate diets, when 3HB becomes an important alternative energy source for the brain. Moreover, 3HB is utilized by the heart and muscle tissues as an energy source, particularly during fasting or prolonged exercise. The kidneys can reabsorb 3HB from the bloodstream and use it as an energy source in the renal tubules [34]. Our results also showed that, compared to liver and heart tissues, the kidney and intestinal tissues of 18-month-old ICR mice exhibited more pronounced signs of aging. This may also be an important reason why 3HB did not exert effects in liver and heart tissues. These findings also provide a reference for the timing of tissue aging within the body.

Cellular senescence and metabolic changes are key drivers of aging [2,35,36,37]. We found that 3HB delays aging by improving both. It ameliorates organ senescence and influences metabolic pathways associated with taurine and hypotaurine metabolism, lipolysis regulation, and central carbon metabolism. Additionally, 3HB reverses age-induced changes in amino acid metabolism, lipid metabolism, and various organ systems. These findings support a new mechanism for 3HB’s anti-aging effects while also providing valuable data on metabolic changes in aging.

Our study identified trigonelline and isoguvacine as metabolites impacted by 3HB and capable of delaying cellular senescence. Trigonelline has previously been shown to extend lifespan and delay age-related diseases in *C. elegans* [38] and improve cognitive function in mice [39]. These findings further support the notion that 3HB delays aging by altering specific metabolites. Our work not only reveals a new mechanism for 3HB but also lays the groundwork for discovering more age-delaying molecules.

However, there are still some limitations in this study. We only used male ICR mice for validation. In the future, we will repeat lifespan studies in female mice and other strains of mice (such as C57BL/6) to assess the variability of genetics and gender. Meanwhile, previous studies have shown that 3HB mimics caloric restriction and fasting and exerts other effects through pathways such as AKT and FOXO [14,40]. This is similar to the mechanisms by which most compounds delay aging. It has also been found that Trigonelline is an NAD precursor [41]. Therefore, the mechanism by which 3HB delays aging may not include killing old cells, and the combination of dasatinib + quercetin may play a role in delaying aging through multiple mechanisms.

## 5. Conclusions

This study reveals the anti-aging potential of 3-hydroxybutyrate (3HB), an endogenous metabolite. The results show that 3HB can delay cellular senescence and extend yeast lifespan, and it also increases the median lifespan of mice by 21.0%, while alleviating age-related tissue and organ decline. Mechanistically, we found that the anti-aging properties of 3HB are mediated by its ability to delay cellular senescence and reprogram metabolism, while also promoting the production of beneficial metabolites such as trigonelline and isoguvacine. Additionally, 3HB has advantages in terms of cost and safety, as it can be obtained from microbial poly-(3-hydroxybutyrate) and is an endogenous metabolite. These findings highlight the promising therapeutic potential of 3HB as an anti-aging intervention and provide new insights into its underlying mechanisms.

## Data Availability

RNA-seq data from kidneys of mice treated with 3HB and control mice (GSE262770). Any additional information required to re-analyze the data reported in this paper is available from the lead contact upon request.

## Supplementary Materials

The following supporting information can be downloaded at: https://www.mdpi.com/article/10.3390/nu17101647/s1, Figure S1. 3HB delays cellular senescence and extends the lifespan of yeast; Figure S2. Effect of 3HB on mouse cardiac function; Figure S3. The influence of 3HB on tissues of aged mice; Figure S4. The influence of 3HB intervention on tissue cell senescence in mice; Figure S5. The influence of 3HB on metabolic changes of aged mice; Figure S6. The influence of 3HB on metabolic changes of aged mice; Table S1.
