# Supplementary material for: Unveiling the Anti-Aging Potential of 3HB: Lifespan Extension and Cellular Senescence Delay
Source: Nutrients. 2025 May 12;17(10):1647. doi: 10.3390/nu17101647 (PMC12114465; doi:10.3390/nu17101647)
Supplement: Supplementary file 1 [file nutrients-17-01647-s001.zip › nutrients-3620402-supplementary Figures.pdf]

nutrients

MDPI

Supplementary Materials

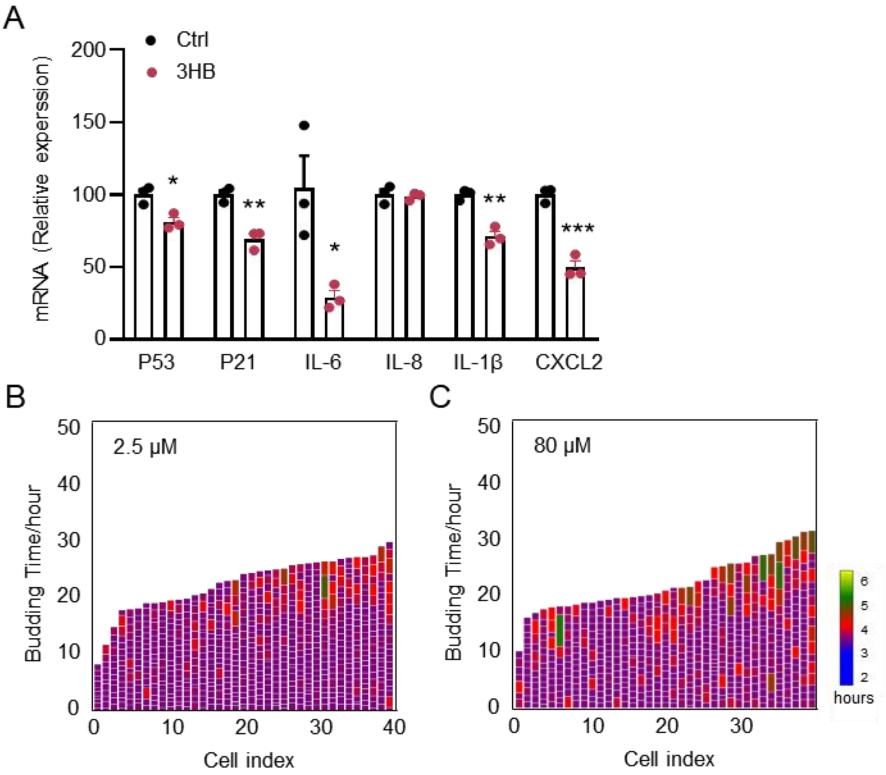

Figure S1. 3HB delays cellular senescence and extends the lifespan of yeast. (A) qPCR detection of the expression levels of P53, P21, IL-6, IL-8, IL-1β, and CXCL2 in senescent 2BS cells (PD45) treated with 10 μM 3HB. (B-C) Germination diagrams of 3HB-treated mother cells show cell cycle duration and heterogeneity (n=40 for each group). (See index color scale. Duration is 1.4 h or less. Cell cycle is colored in the color purple). The *x*-axis shows a single parent cell as a vertical bar, while budding events are shown as white horizontal partitions. Data represent the mean ± SEM. P values were determined by one-way ANOVA, two-way ANOVA, or Student's t-test. (* P<0.05, ** P<0.01, *** P<0.001).

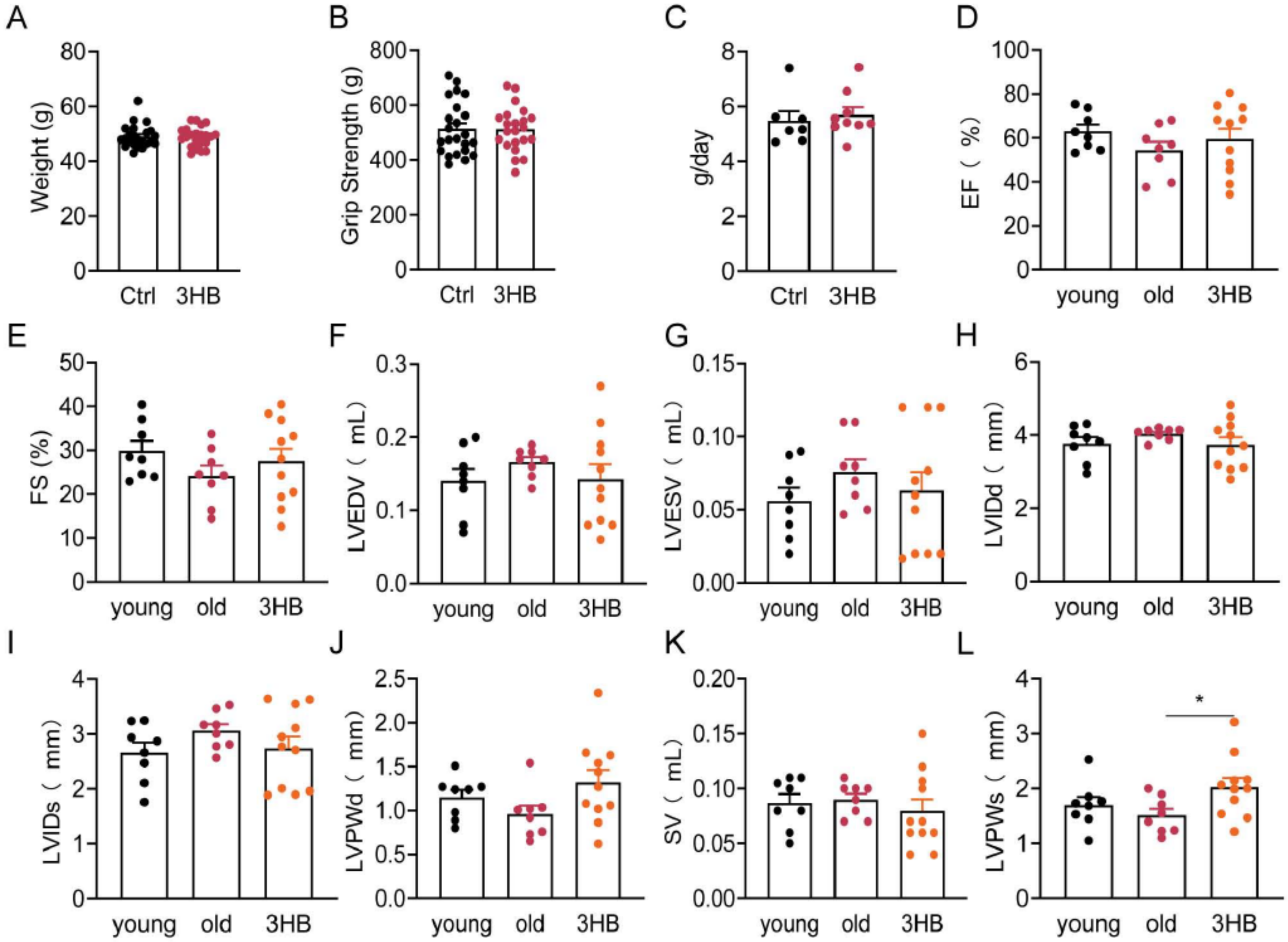

Figure S2. Effect of 3HB on mouse cardiac function. (A-B) Body weight and grip strength of mice before drug administration. (C) Diet of mice at the time of 3HB intervention. (D-L) Effect of 3HB intervention on cardiac function in 20-month-old male ICR mice. Data represent the mean ± SEM ($n \geq 6$). P values were determined by one-way ANOVA, two-way ANOVA, or Student's t-test. (* $P<0.05$, ** $P<0.01$, *** $P<0.001$).

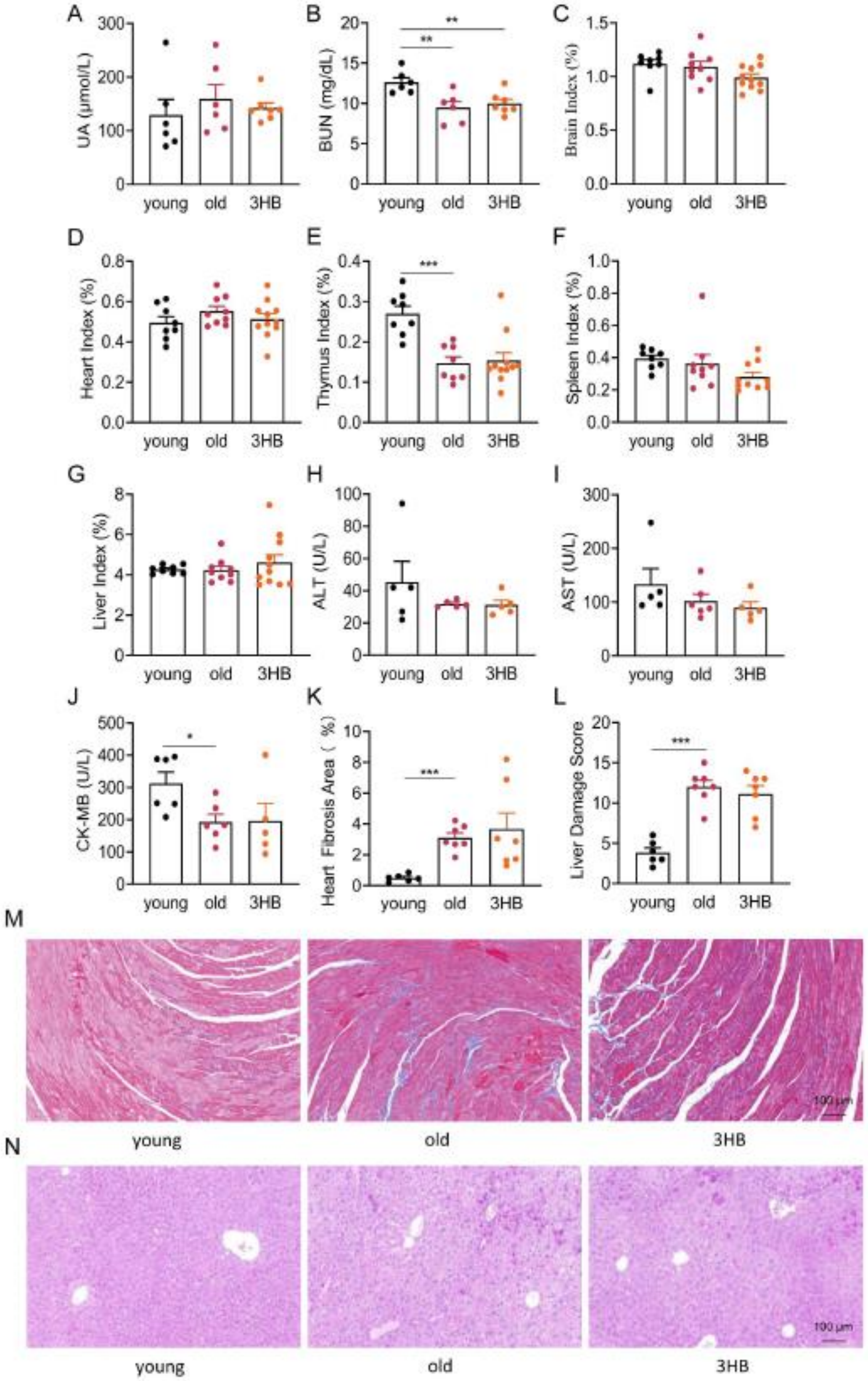

Figure S3. The influence of 3HB on tissues of aged mice. (A-B) Blood biochemical assays of male ICR mice at 2 months (young), 20 months (old), and 20 months treated with 3HB (3HB) were examined for renal function uric acid (UA) and blood urea nitrogen (BUN). (C–G) Brain, heart, thymus, spleen, and liver organ index of male ICR mice at 2 months (young), 20 months (old), and 20 months treated with 3HB (Organ weight/body weight*100%). (H-J) Blood biochemical assays of male ICR mice at 2 months (young), 20 months (old), and 20 months treated with 3HB (3HB) were

examined for liver function and cardiac function, alanine aminotransferase (ALT), aspartate transaminase (AST), and creatine kinase-MB (CK-MB). (K–N) HE and Masson staining to detect the histomorphology of the heart and liver of male ICR mice at 2 months (young), 20 months (old), and 20 months treated with 3HB (3HB), and their quantitative statistics. Data represent the mean ± SEM (n ≥ 6). P values were determined by one-way ANOVA, two-way ANOVA, or Student's t-test. (* P<0.05, ** P<0.01, *** P<0.001).

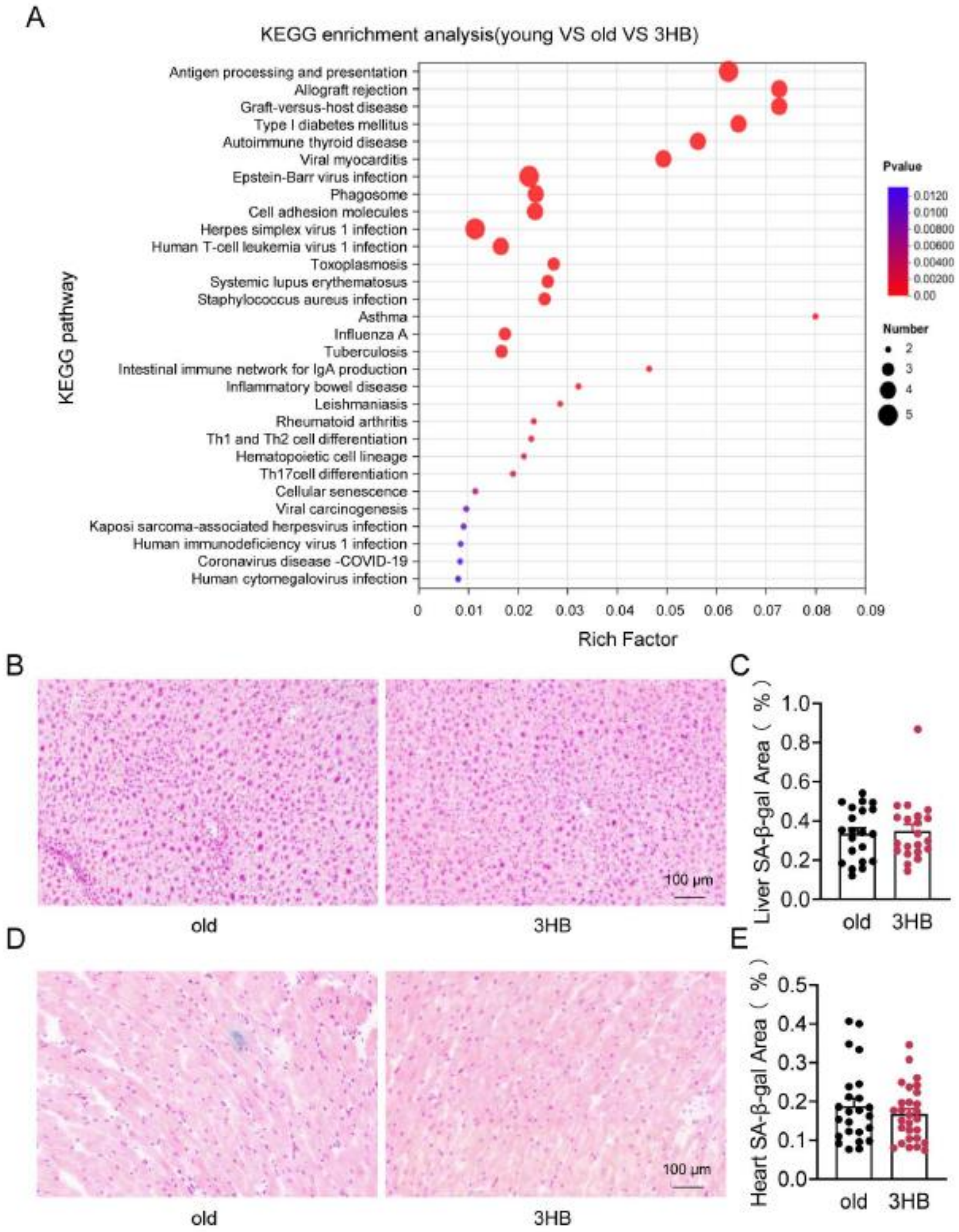

Figure S4. The influence of 3HB intervention on tissue cell senescence in mice. (A) KEGG enrichment analysis of differential genes among the three groups (2-month-old, 20-month-old, and 20-month-old male ICR mice with 3HB intervention) (n=3). (B-C) SA-β-Gal staining to detect the effect of 3HB intervention on the cellular senescence of liver tissues in 20-month-old male ICR mice and statistics (n ≥ 6). (D-E) SA-β-Gal staining to detect the effect of 3HB intervention on the

cellular senescence of heart tissues in 20-month-old male ICR mice and statistics (n ≥ 6). Data represent the mean ± SEM. P values were determined by one-way ANOVA, two-way ANOVA, or Student's t-test. (* P<0.05, ** P<0.01, *** P<0.001).

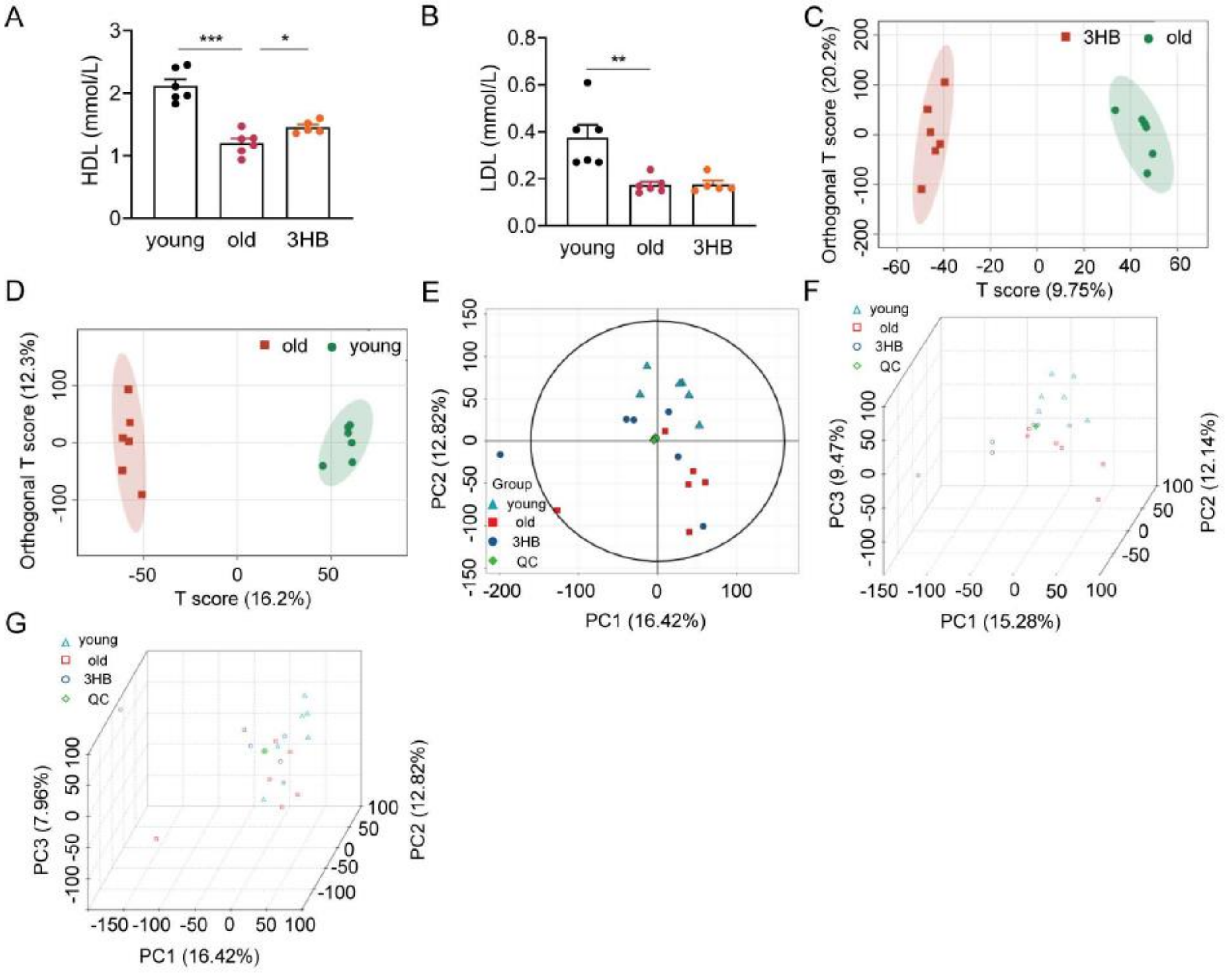

Figure S5. The influence of 3HB on metabolic changes of aged mice. (A-B) Blood biochemistry for high-density lipoprotein (HDL) and low-density lipoprotein (LDL) in male ICR mice at 2 months (young), 20 months (old), and 20 months treated with 3HB (3HB). Metabolomics of feces from 20-month-old ICR mice at 2 months, 20 months (old), and 20 months treated with 3HB (3HB). (C) PCA analysis (POS) of differential metabolites in the aged and 3HB-intervened aged groups. (D) PCA analysis (POS) of differential metabolites between the younger and older groups. (E) PCA analysis of differential metabolites between the three groups (POS). (F-G) PCA analysis (3D) of differential metabolites between the three groups (NEG and POS). Data represent the mean ± SEM (n ≥ 6). P values were determined by one-way ANOVA, two-way ANOVA, or Student's t-test. (* P<0.05, ** P<0.01, *** P<0.001).

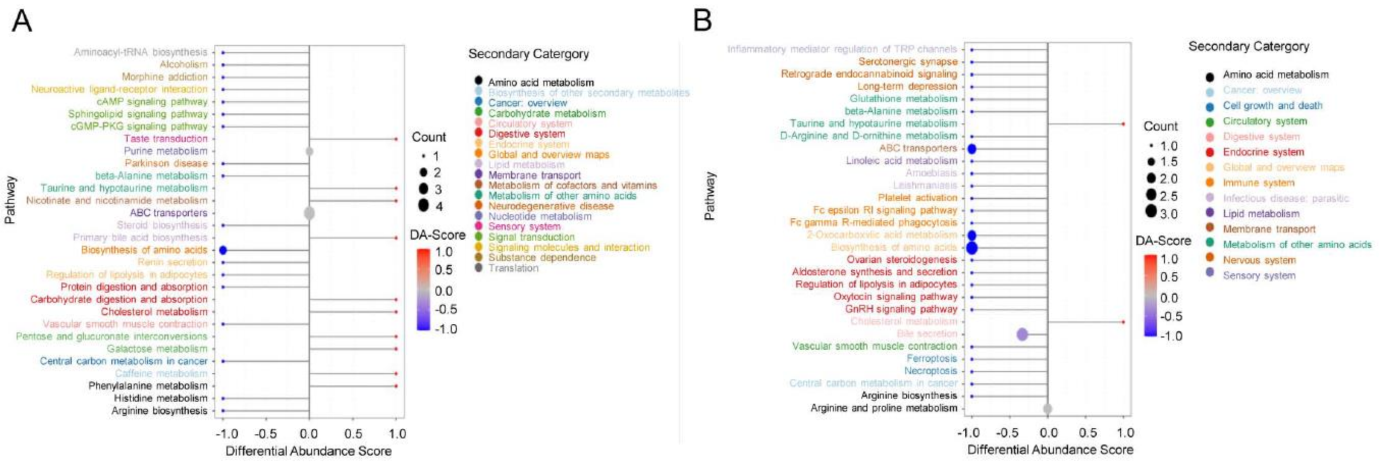

Figure S6. The influence of 3HB on metabolic changes of aged mice. (A) KEGG enrichment analysis of differential metabolites in young and senescent mice. (B) KEGG enrichment analysis of differential metabolites due to 3HB intervention. Data represent the mean ± SEM ($n \geq 6$). P values were determined by one-way ANOVA, two-way ANOVA, or Student's t-test. (* $P<0.05$, ** $P<0.01$, *** $P<0.001$).
